# Improving *in-silico* normalization using read weights

**DOI:** 10.1038/s41598-019-41502-9

**Published:** 2019-03-26

**Authors:** Dilip A. Durai, Marcel H. Schulz

**Affiliations:** 1 Cluster of Excellence on Multimodal Computing and Interaction (MMCI) and Max Planck Insitute for Informatics (MPII), Saarland University, Saarbrücken, Germany; 2 Saarbrücken Graduate School for Computer Science, Saarland University and International Max Planck Research School for Computer Science, Saarland Informatics Campus, Saarbrücken, Germany; 3 Institute for Cardiovascular Regeneration, Goethe University, Frankfurt am Main, 60590 Germany; 4 German Center for Cardiovascular Research, Partner site Rhein-Main, Frankfurt am Main, 60590 Germany

## Abstract

Specialized de novo assemblers for diverse datatypes have been developed and are in widespread use for the analyses of single-cell genomics, metagenomics and RNA-seq data. However, assembly of large sequencing datasets produced by modern technologies is challenging and computationally intensive. *In-silico* read normalization has been suggested as a computational strategy to reduce redundancy in read datasets, which leads to significant speedups and memory savings of assembly pipelines. Previously, we presented a set multi-cover optimization based approach, ORNA, where reads are reduced without losing important k-mer connectivity information, as used in assembly graphs. Here we propose extensions to ORNA, named ORNA-Q and ORNA-K, which consider a weighted set multi-cover optimization formulation for the *in-silico* read normalization problem. These novel formulations make use of the base quality scores obtained from sequencers (ORNA-Q) or k-mer abundances of reads (ORNA-K) to improve normalization further. We devise efficient heuristic algorithms for solving both formulations. In applications to human RNA-seq data, ORNA-Q and ORNA-K are shown to assemble more or equally many full length transcripts compared to other normalization methods at similar or higher read reduction values. The algorithm is implemented under the latest version of ORNA (v2.0, https://github.com/SchulzLab/ORNA).

## Introduction

Due to the advances in next-generation sequencing technologies, it has now become a routine to generate high coverage datasets. A number of *de novo* algorithms have been designed to assemble these datasets which make the study of the whole genome, metagenome and transcriptome possible^1,2^. Most *de novo* assemblers rely on the de Bruijn graph (DBG) as their base data structure. For a given value of *k*, an assembler builds a DBG with nodes as words of length *k* (*k*-mers) and connects two nodes if they have a *k*-1 overlap. The assembly is generated based on various paths in the DBG. Since most of the assemblers store the *k*-mers in shared memory, the memory requirements for assembling large datasets are excessively high^3,4^.

A number of approaches have been developed to reduce the memory and runtime requirement for assemblies. For instance, the genome assembler, Minia^5^ encodes the DBG using a modified version of bloom filters. Howe *et al*.^6^ proposed a method to assemble large metagenomic datasets, where reads are partitioned based on read overlaps and connectivity and each partition is assembled separately. Kim *et al*.^7^ proposed a modification to the Trinity assembler by pre-clustering the input *k*-mers using the MapReduce framework, which works if a suitable infrastructure is available. Techniques such as entropy based compression^8^ can also be used to reduce the memory required to store *k*-mers. But there is a need to handle collisions which might increase the memory usage^9^.

An alternative approach is to remove redundant reads or low quality reads from the input dataset prior to the assembly process. One way to do this is to trim low-quality regions of a read using *e.g*. Phred scores from Illumina sequencers^10,11^. However, this often leads to decreased assembly performance^12,13^ because informative regions might get removed. Further, the issue regarding the high redundancy of modern datasets is not resolved. A popular algorithm “digital normalization” (Diginorm)^14^, which is a part of the khmer package^15^, obtains the abundance of each *k*-mer in the normalized dataset using a min-count-sketch data structure and calculates, for each read, the median of the abundance values of *k*-mers present in the read. If the median is above a user-defined threshold, then the read is rejected otherwise added to the normalized dataset. A similar idea is used within the Trinity assembler package^16^ or with the NeatFreq algorithm^17^. Bignorm, an extension to Diginorm, additionally incorporates a cutoff on the base quality values of reads^18^ and thus combines ideas from Diginorm with quality-based trimming.

All these algorithms run into the risk of losing *k*-mers, which form connections between two sections of a contig resulting in a suboptimal assembly. The ORNA approach addresses this caveat and formulates read reduction as a set multi-cover (SMC) optimization problem^19^. The normalized dataset retains all *k*-mers from the original dataset, thus preserving all the connections of the original DBG (unweighted nodes and edges). Here we describe ORNA-Q and ORNA-K, which extend ORNA with a new *weighted set multi-cover formulation* to include base quality values (ORNA-Q) or *k*-mer abundance values (ORNA-K). Instead of relying on additional cutoffs, as done in Bignorm, we devise a fast greedy algorithm that creates a reduced read dataset, while maximizing read quality or *k*-mer abundance at the same time. On RNA-seq datasets we show that ORNA-Q and ORNA-K reduced datasets lead to more contiguous transcriptome assemblies than ORNA, Diginorm, and Bignorm. ORNA-Q and ORNA-K create reduced datasets of high read quality or *k*-mer abundances, respectively, which may be advantageous for downstream applications. The proposed algorithms are available as parameters in the latest version of ORNA software (v2.0, https://github.com/SchulzLab/ORNA).

## Methods

### Set multi-cover formulation

In ORNA, a normalized dataset is obtained by approximating the minimum number of reads required to retain all *k*-mers from the original dataset. Because we present an extension of ORNA, we will first briefly review that.

Let ${\mathcal R} ={r}_{1},{r}_{2},\,\ldots ,\,{r}_{n}$ be a set of *n* reads of fixed length *s*. Each read *r* consists of *k*-mers (short words of length *k*). A DBG is constructed by creating a node for every *k*-mer and connecting two nodes if they have *k* − 1 bases overlap. For our convenience, we label each edge with a string label *l* of length *k* + 1, which is formed by merging the source vertex with the destination vertex. The source vertex is the prefix of the label and the destination vertex is the suffix of the label. Let *L* = *l*_1_, *l*_2_, …, *l*_*m*_ be the set of all possible labels obtained from ${\mathcal R}$. Since these labels are also generated from the reads in *R*, each read $r\in  {\mathcal R}$ can be considered as a set of labels. It can then be deduced that $\mathop{\cup }\limits_{r\in  {\mathcal R} }r=L$. The goal of ORNA is to find a minimum cardinality subset ${\mathcal R} ^{\prime}$ via solving the following set multi-cover problem^19^:

1 $$\begin{array}{c}{\bf{I}}{\bf{n}}{\bf{s}}{\bf{t}}{\bf{a}}{\bf{n}}{\bf{c}}{\bf{e}}:{\rm{A}}\,{\rm{dataset}}\, {\mathcal R} \,{\rm{of}}\,n\,\mathrm{reads},\,{\rm{a}}\,{\rm{set}}\,{\rm{of}}\,{\rm{labels}}\,L\,{\rm{obtained}}\,{\rm{from}}\, {\mathcal R} \,{\rm{such}}\,{\rm{that}}\\ \,\mathop{\cup }\limits_{r\in  {\mathcal R} }\,r=L\,{\rm{and}}\,{\rm{a}}\,{\rm{threshold}}\,{t}_{l}\ge 1\,{\rm{for}}\,{\rm{every}}\,l\in L.\\ {\bf{V}}{\bf{a}}{\bf{l}}{\bf{i}}{\bf{d}}\,{\bf{s}}{\bf{o}}{\bf{l}}{\bf{u}}{\bf{t}}{\bf{i}}{\bf{o}}{\bf{n}}{\bf{s}}:\, {\mathcal R} ^{\prime} \subseteq  {\mathcal R} \,{\rm{such}}\,{\rm{that}}\,\mathop{\cup }\limits_{r\in  {\mathcal R} }\,r=L\,{\rm{and}}\,\forall l\in L,abund\,(l,\,\, {\mathcal R} ^{\prime} )\,\ge \,{t}_{l}\\ \,{\rm{where}}\,abund\,(l,\,\, {\mathcal R} ^{\prime} )\,{\rm{denotes}}\,{\rm{the}}\,{\rm{number}}\,{\rm{of}}\,{\rm{occurrences}}\,{\rm{of}}\,{l}\,{\rm{in}}\, {\mathcal R} ^{\prime} .\\ {\bf{Objective}}{\boldsymbol{:}}\,\mathop{{\rm{\arg }}\,{\rm{\min }}}\limits_{ {\mathcal R} ^{\prime} }| {\mathcal R} ^{\prime} |\end{array}$$

The constraints denote that the subset ${\mathcal R} ^{\prime}$ contains all labels and that each label *l* occurs at least *t*_*l*_ many times in ${\mathcal R} ^{\prime}$. The threshold *t*_*l*_ is defined as ${t}_{l}=\lceil lo{g}_{b}(abund\,(l, {\mathcal R} ))\rceil$ where $abund\,(l, {\mathcal R} )$ gives the abundance of *l* in the original dataset *R*. We call *t*_*l*_ a label-specific threshold and *b* is the base of the logarithm function controlling the stringency of the thresholds. Larger values of *b* lead to more reduction of reads. This formulation ensures that labels (k + 1-mers along the edges) are kept in a way that depends on the abundance in the full dataset ${\mathcal R}$, which is important because DBG-based assemblers use *k*-mer abundance values to resolve bubbles and tips and to prioritize contigs^3,4^.

ORNA uses a perfect hash function to access k-mer counts in the full dataset ${\mathcal R}$ as well as in the reduced dataset ${\mathcal R} ^{\prime}$. It greedily selects reads from ${\mathcal R}$ containing labels *l* ∈ *L* that have not yet reached the desired abundance level *t*_*l*_ and thus $abund\,(l, {\mathcal R} ^{\prime} ) < {t}_{l}$ (Eq. 1). It ignores the ordering step of the classical greedy algorithm to save memory and runtime.

### Weighted set multi-cover formulation

In this work, we extent ORNA’s SMC by assigning a weight to each read of the input dataset. We obtain a subset ${\mathcal R} ^{\prime}$ of dataset ${\mathcal R}$ which fulfills the constraints of ORNA and at the same time minimizes the overall weight $W( {\mathcal R} ^{\prime} )$ of the dataset. Note that, $W( {\mathcal R} ^{\prime} )$ is the sum of weights of all reads in *R*′. We set the weight of a read in one of the following two ways:

**Base quality aware formulation:** For a given read *r*_*i*_ of length *s*, let ${q}_{i}^{j}$ represent the base quality score of *r*_*i*_ at position *j*. We define the *read quality score qr* as:

2 $$q{r}_{i}=\sum _{j=1}^{s}\,{q}_{i}^{j}.$$

Then, the *phred quality weight qw*_*i*_ can be defined as the inverse of read quality score:

3 $$q{w}_{i}=\frac{1}{q{r}_{i}}.$$

**Label abundance aware formulation:** For a given read *r*_*i*_ of length *s*, let ${l}_{i}^{j}$ be label (*k* + 1-mer) starting at position *j* in *r*_*i*_. The abundance of the label ${l}_{i}^{j}$ in the original dataset is represented as ${a}_{i}^{j}$. Let ${a}_{i}=({a}_{i}^{0},{a}_{i}^{1},\,\mathrm{...}\,{a}_{i}^{(s-k)})$ be the set of abundances of labels in *r*_*i*_. We define the *read abundance score kr*_*i*_ as:

4 $$k{r}_{i}=median({a}_{i}\mathrm{).}$$

We then define the *label abundance weight kw*_*i*_ as

5 $$k{w}_{i}=\frac{1}{k{r}_{i}}.$$

We define the *overall read weight* $W( {\mathcal R} )$ for a read dataset ${\mathcal R}$ either as:

6 $$W( {\mathcal R} )=\sum _{{r}_{i}\in  {\mathcal R} }\,q{w}_{i}$$

or

7 $$W( {\mathcal R} )=\sum _{{r}_{i}\in  {\mathcal R} }\,k{w}_{i}.$$

Because we have a weight defined for each read, we can extend the SMC problem to a *weighted set multi-cover (WSMC) problem*:

8 $$\begin{array}{c}{\bf{I}}{\bf{n}}{\bf{s}}{\bf{t}}{\bf{a}}{\bf{n}}{\bf{c}}{\bf{e}}:{\rm{A}}\,{\rm{dataset}}\, {\mathcal R} \,{\rm{of}}\,{n}\,\mathrm{reads},\,{\rm{a}}\,{\rm{set}}\,{\rm{of}}\,k+1-{\rm{mers}}({\rm{defined}}\,{\rm{as}}\,{\rm{labels}})L\\ {\rm{obtained}}\,{\rm{from}}\, {\mathcal R} \,{\rm{such}}\,{\rm{that}}\,\mathop{\cup }\limits_{r\in  {\mathcal R} }r=L\,{\rm{and}}\,{\rm{a}}\,{\rm{threshold}}\,{t}_{l}\ge 1\,{\rm{for}}\,{\rm{every}}\,l\in L.\\ {\bf{V}}{\bf{a}}{\bf{l}}{\bf{i}}{\bf{d}}\,{\bf{s}}{\bf{o}}{\bf{l}}{\bf{u}}{\bf{t}}{\bf{i}}{\bf{o}}{\bf{n}}{\bf{s}}: {\mathcal R} ^{\prime} \subseteq  {\mathcal R} \,{\rm{such}}\,{\rm{that}}\,\mathop{\cup }\limits_{r\in  {\mathcal R} }r=L,\,\forall l\in L,\,abund(l,\, {\mathcal R} ^{\prime} )\ge {t}_{l}\\ {\bf{Objective}}:\mathop{{\rm{\arg }}\,\,{\rm{\min }}}\limits_{ {\mathcal R} ^{\prime} }W( {\mathcal R} ^{\prime} )\end{array}$$

From the set of valid solutions, we want to pick a solution which has the minimal read weight $W( {\mathcal R} ^{\prime} )$ (Eq. (8)) but at the same time preserves all labels from the original dataset a certain number of times. As the WSMC problem is a generalization of the SMC problem with weights set equal to one, it follows that the WSMC problem is also NP-hard^20,21^.

### ORNA-Q and ORNA-K

Here we suggest extensions of ORNA named ORNA-Q and ORNA-K providing a solution to the WSMC problem. Finding approximation algorithms for the WSMC problem is a current area of research in computational geometry^20^. The following greedy approach is common for solving the WSMC problem: In the initial state, each element of the universe is treated as *active*, *i.e*., it has not been selected by any of the output sets a certain number of times. Each set in the instance has a weight associated with it. The algorithm iterates over the sets and selects a set which has both the maximum number of active elements and the minimum weight. Here, the universe is the set of all labels in *R*. As mentioned above, each read in *R* is considered as a set of labels and has a weight associated with it. In the classical greedy approach, we have to maintain a data structure holding all reads in an order starting from the read which has both the highest number of active labels and the minimum weight. This order has to be updated after every read selection. Hence, for a dataset of *n* reads, each with *m* labels, this greedy algorithm would take ${\mathscr{O}}({n}^{2}mlog(nm))$ time, which is inefficient for large datasets.

Thus, we follow a simplified version of the greedy algorithm ordering the reads only once, ignoring the reordering of reads after every selection. We use two different counting sort based strategies using the weights defined above:

**Phred quality based weight (ORNA-Q):** For a dataset ${\mathcal R}$ with *n* reads each of length *s*. Let Σ denote the set of all characters of the phred based quality scores. Then ${\mathscr{C}}={{\rm{\Sigma }}}^{s}$ denotes all possible combinations of the phred scores for a read. For each such combination, we can compute the corresponding read quality score *qr*. The value with the largest possible read quality score is denoted as *qr*_*max*_.

For each read ${r}_{i}\in  {\mathcal R}$ we compute its *qr*_*i*_, ranging from 0 to *qr*_*max*_. We then initiate an array *T* of size *qr*_*max*_. Every read quality score obtained by any combination in ${\mathscr{C}}$ can be mapped to an index of *T*. *T* is then used to record the number of times a particular read quality score is observed in ${\mathcal R}$. In other words, *T*[*qr*_*i*_] records the number of times *qr*_*i*_ is encountered in ${\mathcal R}$.

**Label abundance weight (ORNA-K):** Given a dataset ${\mathcal R}$ consisting of *n* reads, we first calculate *kr*_*i*_ for each read ${r}_{i}\in  {\mathcal R}$. The reads are then divided into *d* bins of size *b* based on their read abundance score. We maintain an array *T* of size *d*. Each index *i* in *T* represents a bin and *T*[*i*] records the number of elements in bin *i*. For instance, if the size of each bin is 1000, then say *T*^10^ contains the number of reads having label abundance weight between 9000 and 10000.

We then proceed by performing a counting sorting step to sort reads by their read weights^22^. Briefly, an array ${\mathcal R}$ of size *n* is created to store the ordered list of reads. This array is divided into *f* chunks, where *f* is the number of non-zero entries in *T*. The algorithm iterates over the dataset ${\mathcal R}$ and calculates the read weight for each read ${r}_{i}\in  {\mathcal R}$. The sorted position *pos* of *r*_*i*_ in ${\mathcal R}$ is computed using *T* and stored in the corresponding chunk. To reduce the memory requirement of the algorithm, the chunks are stored on disk as files and are combined to a single file containing the ordered list of reads.

The greedy read selection is applied to the sorted list of reads. We iterate sequentially over the list of reads. We maintain a counter which records the abundance of a label in the accepted dataset. For a given read, we check whether there is a label present in the read, which has not been covered *t* times in ${\mathcal R} ^{\prime}$. If such a label is present then the read is added to ${\mathcal R} ^{\prime}$ otherwise it is rejected. In ORNA-Q, since the input set is sorted on the basis of phred scores, each iteration will process one of the reads, which has the lowest weight in the remaining set. In ORNA-K, each iteration would process a read which has the lowest or close to the lowest weight, since the reads are sorted into a bin based on label abundances and the ordering within the bin is ignored.

**Normalization of paired-end data:** For paired-end datasets, normalization is run in three stages. In the first stage, for each pair in the dataset, we first calculate the *pair-score*, which is the sum of the individual scores of each read in the pair. Similar to single read data, we take the inverse of the *pair-score* to obtain the final *pair-weight*. We then proceed by sorting the pairs according to the *pair-weights*. In the second stage, we iterate over the sorted pairs sequentially and accept the pair only if both reads of the pair contain at least one *k*-mer which has not been covered *t* times in ${\mathcal R} ^{\prime}$, which is our acceptance condition. If only one read satisfies the acceptance condition, then the pair is considered as *marked*, otherwise the pair is rejected. The count for the *k*-mers for marked pairs are not incremented. In the final stage, all the marked reads are iterated in the order in which they were first encountered in the sorted dataset and a pair is accepted if one of the reads satisfies the acceptance condition.

### Runtime

Given a dataset *R* consisting of *n* reads, the counting sort step in ORNA-Q runs in linear time ${\mathscr{O}}(ns)$ where *s* is the average length of reads in the dataset. The counting sort step of ORNA-K runs in ${\mathscr{O}}(nm)$ time where *m* is the number of labels in a read. The worst-case runtime of ORNA-Q and ORNA-K is dominated by the DSK *k*-mer counting which takes ${\mathscr{O}}(nm\,log\,(nm))$ time^23^. All other steps, i.e, streaming through the reads and storing accepted reads, take ${\mathscr{O}}(n)$ time.

### Data retrieval

Two different RNA-seq datasets were used for the analysis: 147M paired-end (PE) reads from brain tissue^24^ (SRR332171) and 216M PE reads from HeLa cell line (SRR317049). All datasets were downloaded from the SRA run browser.

### Transcriptome assembly and evaluation

For comparing the performance of ORNA-Q/-K, we normalized the datasets using ORNA (version 0.2), Diginorm (version 2.0) and Bignorm (version 0.01). We varied the base *b* parameter of ORNA and ORNA-Q/-K to achieve different amounts of reduction. For Diginorm and Bignorm, we varied the coverage cutoff and quality score cut-off parameter respectively. We show the values in Supp. Table S1. The *k*-mer parameter for all the algorithms was set to *k* = 22. Additional parameter required by Diginorm is the hash table size, which was set to 32e + 8.

We assembled all the reduced datasets using the DBG-based *de novo* assembler TransABySS^25^(version 1.5.3). The obtained assemblies were evaluated using REF-EVAL (^26^, version 1.11) which we explain briefly. REF-EVAL aligns the reads to ENSEMBL transcripts, downloaded from ENSEMBL^27^ (version 65) to estimate a *true assembly*. True assemblies are regions in the annotated transcripts, which are overlapped by aligned reads. It then bidirectionally aligns the *true assemblies* and the *de novo* assemblies using blat^28^ (version 36) and calculates nucleotide precision, recall and F1 scores. As mentioned above, we generated different assemblies for datasets, obtained by varying the parameters of the normalization algorithms. For each normalization algorithm and dataset, we obtained the mean F1 score of all assemblies. The assembly contiguity was measured by aligning the assembled transcripts to a reference genome using blat. We then match the overlap against a set of annotated Ensembl transcripts. The number of Ensembl transcripts that were fully assembled by at least one distinct transcript was termed as *full-length transcripts*.

### Evaluation of reads

In order to evaluate reads from a complete or partial dataset ${\mathcal R}$, we introduce the average read quality score (${\bar{Q}}_{ {\mathcal R} }$) and average read abundance score (${\bar{K}}_{ {\mathcal R} }$) as:

9 $${\bar{Q}}_{ {\mathcal R} }=\frac{\sum _{{r}_{i}\in  {\mathcal R} }q{r}_{i}}{ {\mathcal R} |},\,{\bar{K}}_{ {\mathcal R} }=\frac{\sum _{{r}_{i}\in  {\mathcal R} }\,k{r}_{i}}{| {\mathcal R} |}.$$

## Results

### Read ordering affects *in-silico* normalization

*In-silico* normalization methods like Diginorm^14^ and ORNA^19^ select reads by going through the read file in the order created by the sequencer. Diginorm goes over the dataset and selects a read if its median *k*-mer abundance is below a certain threshold. ORNA, on the other hand, considers normalization as a SMC problem. It considers the set of all *k*-mers present in the dataset as universe and each read as a set of *k*-mers. It then proceeds by selecting the minimum cardinality sub-collection of reads required to cover all the *k*-mers from the original dataset a certain number of times (Eq. 1). Most erroneous reads have base(s) with low phred scores and are likely to have lower abundant *k*-mers at these positions, although the latter must not be true for non-uniform coverage data as in RNA-seq and metagenomics^12^. We hypothesize that incorporating these features into the formulation of ORNA would improve the greedy selection of the reads and result in a normalized dataset containing less sequencing errors.

**Figure 1:**
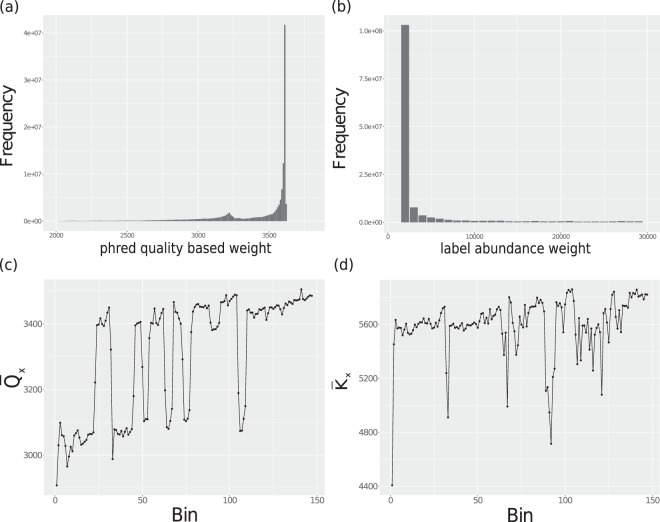
Average read quality score (**a**) and average read abundance score (**b**) distribution in Brain RNA-seq data. The position-wise distribution of average read quality score (**c**) and average read abundance score (**d**) in the brain dataset. Reads in both datasets were divided into bins of 1 million (x-axis). These bins were then considered as partial datasets and the scores (${\bar{Q}}_{x}$ and ${\bar{K}}_{x}$) was calculated for each bin (y-axis).

One way to incorporate these features is to remove all reads which have either a low phred quality weight or low abundance weight. But this may remove connectivity in the DBG. In this work, we suggest a measure for each read in terms of *read quality/abundance score*. We set the read score for *r*_*i*_ in either of the following two ways-(1) by taking the sum of phred scores of all the bases in *r*_*i*_ and terming it as read quality score *qr*_*i*_ (Eq. 2) or (2) by taking the median of abundances of all labels present in *r*_*i*_ and terming it as read abundance score *kr*_*i*_ (Eq. 4). In Fig. 1a,b, we show the distribution of both types of scores in the brain dataset. We observe that there is a considerable percentage of reads which either have a low quality or have a low abundance. Many of these low scoring reads may not get used by the assembler. Next, we looked into positions of these low scoring reads in the dataset. We divided the reads into bins of size one million according to their position in the Fastq file. For each bin *x*, we calculated the average phred quality score ${\bar{Q}}_{x}$. Figure 1c,d show the position-wise distribution of average read quality scores and average read abundance scores in the brain RNA-seq dataset. In case of average read quality scores, we see that many low scoring (potentially erroneous) reads are at the beginning of the file. Towards the end of the dataset, there is an enrichment of reads having high read quality scores. In case of label abundance scores (Fig. 1d), there is a decent percentage of high abundance reads towards the end of the dataset, but it is obvious that both types of scores do not behave exactly similar. In general, high scoring reads are worth keeping, but since ORNA traverse the dataset sequentially, there is a chance that many low scoring reads are included, and thus a suboptimal selection of reads is obtained. Hence, considering the read quality or read abundance score during optimization appears useful.

**Figure 2:**
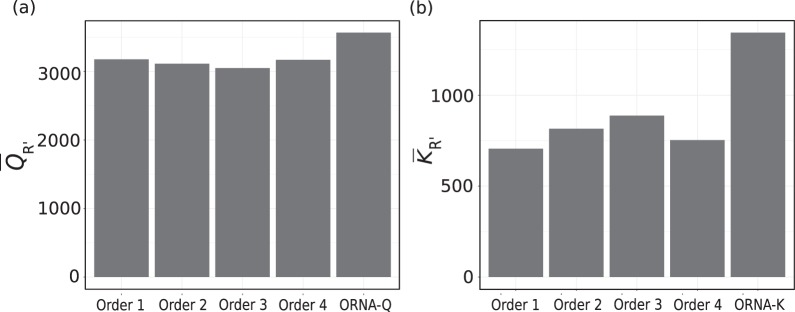
Comparison of ORNA-Q (**a**) and ORNA-K (**b**) against ORNA applied on different read orderings (x-axis) for the brain dataset. *Order 1* denotes the original dataset ordering. *Order 2–4* was obtained by random reshuffling of the reads. The average scores of the reads from the reduced dataset is shown on the y-axis. All the above orders results in similar amount of reduction.

**Figure 3:**
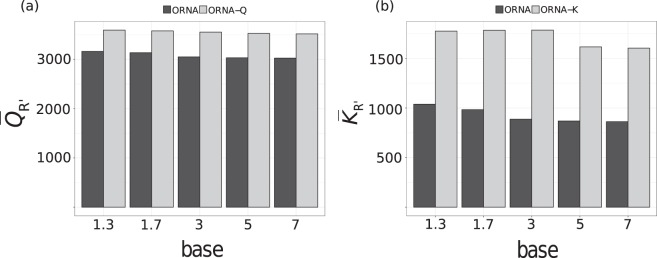
Effect of varying the log base parameter b (x-axis) on the average read weight (y-axis), (**a**) $\bar{Q}(R^{\prime} )$ and (**b**) $\bar{K}(R^{\prime} )$ of the normalized brain datasets. The black and grey bars represent normalization using ORNA-Q/-K and ORNA, respectively.

In this work, we propose ORNA-Q and ORNA-K, weight-based heuristic approaches, which extend the optimization function of ORNA. ORNA-Q/-K assign a weight to each read in the dataset which is the inverse of either read quality score (ORNA-Q) or the read abundance score (ORNA-K). ORNA-Q/-K minimize the overall weight of the normalized dataset in such a way that all labels of the original dataset are covered a certain number of times. In turn, this means that ORNA-Q/-K maximize the overall score of the reduced dataset (Eq. 8, see Methods). We achieve this by reordering the input set of reads according to read scores and proceed with the read normalization on the ordered set as input. In Fig. 2a,b, we compare the performance of ORNA-Q and ORNA-K respectively against ORNA for the brain dataset in terms of reduction and the average score of normalized datasets. We applied ORNA on four different read orderings *Order 1–4* and compared that to the optimization with ORNA-Q and ORNA-K. *Order 1* is the original ordering in the Fastq file. *Orders 2–4* were generated by random reshuffling of the reads. The average read quality score ${\bar{Q}}_{R^{\prime} }$ and average read abundance score ${\bar{K}}_{R^{\prime} }$ were calculated for each of the normalized datasets. As can be seen, the ordering of the reads influences the number of reads reduced as well as the average score obtained. We observed that both ORNA-Q and ORNA-K improve the average read score in the reduced datasets as compared to all other orderings. Next, we wanted to investigate the effect of the base *b* of the logarithmic function on the average read weight obtained by ORNA-Q and ORNA-K. Figure 3a,b compares the obtained average read scores for all versions of ORNA on the brain datasets for several parameters. ORNA, ORNA-Q, and ORNA-K traverse the dataset sequentially. Since ORNA-Q and ORNA-K sort the dataset before normalization, they keep more high scoring reads than ORNA. We observed that the average read score of ORNA-Q and ORNA-K normalized datasets are constantly higher and behave more stable than for ORNA normalized datasets.

### Comparison of assembly performance

A read normalization algorithm should normalize a dataset with little effect as possible on the quality of the assemblies produced from them. To evaluate the quality of the assemblies produced from ORNA-Q/-K we used REF-EVAL program from the Detonate package^26^. REF-EVAL compares nucleotides in the assemblies with that in a reference and calculates nucleotide level precision, recall and F1 score (see Methods). The nucleotide F1 scores, nucleotide precision, and recall were similar and stable across all normalization algorithms. We show the average F1 score, average nucleotide precision and average nucleotide recall obtained from assemblies using various normalization parameters in Table 1. We observed that nucleotide precision and recall have a variable behavior across datasets and across algorithms. For instance, for the Brain dataset, assemblies generated from ORNA-Q reduced sets have a better mean recall value across algorithms, whereas precision was highest for Bignorm. In the case of F1 scores, ORNA-Q and ORNA-K have a slightly better F1 score as compared to other normalization algorithms in all the cases. It is interesting to note that, both ORNA-Q and ORNA-K have better F1 than ORNA. This might be due to the retention of reads having highly abundant *k*-mers and reads of higher average quality. These reads are less likely to be errors and hence generate better assemblies. Although the F1 score is a widely used estimate for assembly quality, it does not measure the contiguity of an assembly.

**Table 1:** Comparison of mean F1 scores, nucleotide precision, and nucleotide recall.

Dataset	measure	Unreduced	ORNA-K	ORNA-Q	ORNA	Diginorm	Bignorm
Brain	*F*_1_ score	0.442	0.441	**0.442**	0.438	0.441	0.426
HeLa	*F*_1_ score	0.280	**0.280**	0.279	0.278	0.273	0.272
Brain	Recall	0.347	0.347	**0.350**	0.345	0.349	0.331
HeLa	Recall	0.354	0.355	0.360	0.359	**0.372**	0.369
Brain	Precision	0.610	0.608	0.603	0.603	0.598	**0.635**
HeLa	Precision	0.232	**0.232**	0.227	0.227	0.214	0.219

Brain and HeLa datasets normalized by the five algorithms (ORNA, ORNA-Q/-K, Diginorm, and Bignorm) were assembled using TransABySS. Several normalized datasets were obtained by varying parameters for each algorithm. Each of these datasets was assembled separately. All the assemblies were then evaluated using REF-EVAL. Averages were taken over results obtained from different assemblies. The mean F1, precision and recall scores obtained for the original (unreduced) dataset is shown in the first column. The highest mean obtained by any normalization algorithm is shown in bold.

**Figure 4:**
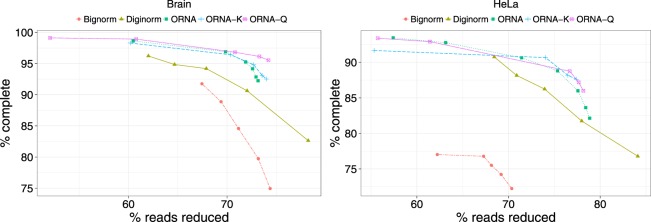
Comparison of assemblies generated from normalized datasets. The % *of reads reduced* (x-axis) by a normalization algorithm is compared against % *of complete* (y-axis: an assembly performance measure). Each point on a line corresponds to a different parametrization of the algorithms. (**a**,**b**) Represent TransABySS assemblies (k = 21) applied to normalized brain and HeLa data, respectively.

To measure this, we obtained the number of *full-length transcripts* by aligning the assemblies to a reference genome and comparing the alignment against existing gene annotations (see Methods). We considered the total number of *full-length* transcripts obtained by running the assembler on the original unreduced dataset as *complete*. We then compared the normalization algorithms in terms of *% of complete* as done in ORNA^19^. We compared the assembly performance of ORNA-Q and ORNA-K against the performance of ORNA, Diginorm, and Bignorm for brain and HeLa datasets. We varied the parameters of the normalization algorithms (base parameter for ORNA-Q/-K and ORNA, coverage parameter of Diginorm and Quality cutoff of Bignorm, see Methods). Figure 4a,b compare the amount of reads reduced (x-axis) against *% of complete* (y-axis) for the brain and HeLa datasets, respectively. We observe that as more reads are reduced, the quality of assembly degrades for all algorithms. For the brain dataset, ORNA-Q and ORNA-K are constantly able to retain a higher number of full-length transcripts as compared to the other algorithms tested.

ORNA-Q and ORNA-K always perform better or as good as ORNA, which suggests that optimizing the read quality score or *k*-mer abundances during reduction has a positive impact on assembly contiguity. For the brain dataset, we see that both ORNA-Q and ORNA-K perform similar to ORNA for lower reduction values (55–75%). But at a higher percentage of reduction (above 75%), we see that both ORNA-Q and ORNA-K retain more *full-length* transcripts than ORNA. One reason for this may be the read ordering in the brain fastq file (Fig. 1c). Most of the low quality reads are found in the first half of the dataset. There is a considerable fraction of lowly abundant reads in the first half (Fig. 1c). For high values of reduction, *i.e*, for higher values of the log base *b*, a significantly smaller set of reads is necessary to keep enough copies of observed *k*-mers. Hence, when the phred scores of the bases or the *k*-mer abundances are taken into consideration, the ORNA-Q normalized dataset consists only of reads having bases of high phred score and ORNA-K reduced datasets only contain reads with high abundant *k*-mers, which appears to result in an improved assembly. In case of lower percentage of reduction (55–60%), ORNA and ORNA-Q/-K retain a similar set of reads from the original file resulting in similar assembly performance. For HeLa, the performance of ORNA-Q and ORNA-K are only slightly better than ORNA at higher reduction percentage. This may be due to the ordering of reads in the HeLa dataset (Supp. Fig. S1). Different to the brain dataset, many high-quality reads are already at the beginning and the reads with lowest average qualities are found at the end. The slight improvement of ORNA-K and ORNA-Q might be due to low scoring reads at the beginning of the dataset, which are pushed towards the end by the sorting process and hence not selected by ORNA-Q and ORNA-K. This supports the idea that the position of high-quality reads in the dataset influences *in-silico* normalization algorithms. But ORNA-Q, ORNA-K and ORNA perform better than Diginorm and Bignorm mainly due to the retention of all the *k*-mers from the original dataset. Bignorm, showed the largest loss in assembly performance for high levels of read reduction, which are with strongest quality thresholds. This suggests that *a priori* filtering of lower quality reads is too stringent and discards reads that form important connections in the assembly graph. Similar results have been observed for quality-based read trimming and RNA-seq assembly^13^.

### Comparison of resource requirements

The runtime and the memory consumption of ORNA-Q and ORNA-K were compared against the resource consumption of Bignorm and Diginorm. For all the algorithms, the *k*-mer size was set to 22. Like ORNA, ORNA-Q, and ORNA-K store the *k*-mers from the dataset in bloom filters which is memory and runtime efficient. We show the comparison of the resources required by ORNA-Q and ORNA-K against those required by ORNA, Diginorm, and Bignorm in Table 2. For comparison, we chose normalization parameters for all the algorithms such that the normalized datasets obtained from each algorithm have similar numbers of reads. All the algorithms were run on a machine having Intel Xeon CPU E7-8837 processor and having 1 TB RAM.

**Table 2:** Runtime (in minutes) and memory (in GB) required by ORNA-Q/-K, ORNA, Diginorm and Bignorm for normalizing Brain (147 M) and HeLa dataset (216 M).

Method	Brain (147 M–35.1 GB)			HeLa (216 M–60.7 GB)		
	% reduced	time [min]	mem [GB]	% reduced	time [min]	mem [GB]
ORNA	69.8	112 (42)	6.38 (6.31)	75.31	219 (64)	9.81 (9.85)
ORNA-Q	70.65	116 (50)	7.10 (7.13)	72.72	223 (70)	10.01 (10.02)
ORNA-K	70.3	130 (52)	6.41 (6.5)	73.86	279 (75)	9.98 (10.01)
Diginorm^*a*^	72.03	135	12.5	72.91	198	12.51
Diginorm^*b*^	70.51	112	6.26	75.10	155	9.76
*Bignorm^*a*^	69.38	(47)	41.94	71.28	(58)	41.94
Bignorm^*b*^	69.39	(41)	5.23	71.26	(55)	5.24

*Notes:* The memory required to store the complete dataset in the main memory is indicated in brackets next to the name of the dataset. The column *% reduced* states the percent of reads reduced by each method. Time and memory as obtained by running the algorithm with 10 threads (if possible) are shown in brackets. *Bignorm always runs with 4 cores and fixed memory settings.

Both Diginorm and Bignorm use the count-min sketch for counting *k*-mers. The default version of Bignorm fixes 40 GB of RAM for the data structure. In both the algorithms, users can control the memory consumption by tuning the algorithm parameters, but the amount of memory used in both the algorithms is directly proportional to the precision of *k*-mer counts. Hence, reducing the memory consumption of both Diginorm and Bignorm results in more false positives. Here, we have used two versions of Bignorm (Bignorm^*a*^ and Bignorm^*b*^) and Diginorm (Diginorm^*a*^ and Diginorm^*b*^). Bignorm^*a*^ uses the default setting except the *k*-mer size. In Bignorm^*b*^, we reduced the size of each hash function of the count-min sketch to limit the memory consumed. Similarly, Diginorm^*a*^ uses the default settings (except the *k*-mer size) where the number of hashes is set to 4. Diginorm^*b*^ sets the number of hashes to 2 to reduce the memory requirements (see Supp. Table S2). The parameters for Bignorm^*b*^ and Diginorm^*b*^ were set in a way so that the memory consumption of these variants were similar to that of ORNA-Q and ORNA-K.

We see that ORNA-Q and ORNA-K use a bit more memory and runtime as compared to ORNA, which is required to calculate the read weights and sorting the reads. We observed that the fastest configuration of Bignorm is always slightly faster than ORNA-Q or ORNA-K and that Diginorm is the slowest of the tested methods, mostly because it does not support multithreading. We found that the default memory consumption of Bignorm is huge, but when accepting more false positives it can be reduced significantly.

## Discussion

ORNA normalizes a dataset by keeping a minimum number of reads required to retain all *k*-mers from the original dataset. ORNA processes the dataset in the order obtained by the sequencer. Here, we propose ORNA-Q and ORNA-K which extend the formulation of ORNA to a *Weighted Set Multi Cover (WSMC)* problem by including base qualities or kmer abundances. ORNA-Q/-K fulfill the constraints of ORNA, but at the same time minimizes the overall score of the reduced dataset. We compare the performance of ORNA-Q/-K against ORNA and other normalization algorithms - Diginorm and Bignorm. We show that using the base quality values and the *k*-mer abundances leads to reduced datasets with higher average read quality and often improved *de novo* assembly.

For comparison with Diginorm and Bignorm we used the approach introduced previously^19^. The parameters of the different algorithms led to different read reduction values in a data-dependent manner. Therefore, it is not possible to compare two read reduction methods with fixed parameters on different datasets. Hence, we varied the parameters of each algorithm and compared the assemblies generated by the resultant normalized datasets. We first evaluated the assemblies using REF-EVAL, which provides us nucleotide precision, recall and an F1 score. In general, we found that the assemblies generated by ORNA-Q and ORNA-K were slightly better than the rest of the algorithm. In terms of *full length* assemblies, ORNA-Q and ORNA-K were performing better than ORNA for most of the cases. At higher percentages of reduction, we found that the performance of ORNA-Q and ORNA-K were always better than Bignorm and Diginorm. This might be due to the combined fact that Diginorm and Bignorm loose important *k*-mer information and also is more probable to keep low quality reads as compared to ORNA-Q and ORNA-K. In the Bignorm paper^18^, one parametrization of Diginorm was compared to several parametrizations of Bignorm with varying base quality thresholds. In our experiments on human RNA-seq data Diginorm performed often better or as good as Bignorm. This may be due to several reasons. Bignorm was tested on bacterial single cell datasets, which are likely to show other characteristics than the more complex human transcriptome datasets we analyzed. Also, it should be noted, that Bignorm has three other parameters that affect the decision of whether a read is discarded (denoted as rarity, contribution and abundance threshold). How these parameters affect the performance of Bignorm was not discussed in the Bignorm manuscript or manual. In our comparison, we varied the base quality threshold (−Q) as was done in the Bignorm paper. However, additional optimization of these parameters may lead to better performance on the human RNA-seq datasets we analyzed. In this light, ORNA-Q/-K have the advantage to use base quality values or *k*-mer abundance values without any additional cutoff to set by the user, except the logarithm *b* for computing the *k*-mer thresholds. However, the algorithm we propose is a heuristic and is unlikely to find the optimal solution. Developing efficient approximation algorithms that find better solutions is a topic of further research.

We have shown before that error correction before sequencing is useful to improve the assembly results^19^. This should be done with methods that are tailored for RNA-seq data, such as SEECER^12^ or Rcorrector^29^. Combining both aspects, non-uniform error correction and read normalization, is thus another useful topic for future research.

ORNA-Q and ORNA-K are fast *in-silico* normalization methods that use base quality values or *k*-mer abundances to reduce datasets without the additional need for parametrization and can thus be easily integrated into assembly workflows. Both algorithms are available as parameters in the latest version of ORNA software (v2.0, https://github.com/SchulzLab/ORNA).

## Supplementary information

Supplementary PDF file
