# Supplementary material for: Improving in-silico normalization using read weights
Source: Sci Rep. 2019 Mar 26;9:5133. doi: 10.1038/s41598-019-41502-9 (PMC6435659; doi:10.1038/s41598-019-41502-9)
Supplement: Supplementary file 1 — Supplementary PDF file [file 41598_2019_41502_MOESM1_ESM.pdf]

Supplementary material for the manuscript:
Improving *in-silico* normalization using read weights

Dilip A Durai and Marcel H Schulz

Normalization parameters

Table S1: Parameters used for different normalization algorithms. ORNA-Q, ORNA-K and ORNA requires base b of the logarithm function. Diginorm and Bignorm requires a coverage cut-off value c and quality score cutoff q respectively. The first column contains the datasets used for the algorithms. A star next to the value indicates the default coverage/base of the algorithm.

Dataset	ORNA-K(b)	ORNA-Q(b)	ORNA(b)	Diginorm(c)	Bignorm(q)
Brain	(1.7*,3,5,7,9)	(1.3,1.7*,3,5,7,11)	(1.3,1.7*,3,5,7,9)	(5,10*,15,20,25)	(5,10,15,20*,25)
HeLa	(1.3,1.7*,3,5,7)	(1.3,1.5,5,7,9)	(1.3,1.5,2,3,5,7,9)	(5,10*,15,20,25)	(1,5,10,15,20*)

Table S2: Command lines used for Bignorm and Diginorm for calculation of memory and runtime requirements.

Algorithm	Input Dataset	Command
Bignorm[a]	Brain(SRR332171)	Bignorm -Q 10 -k 22
Diginorm[a]	Brain(SRR332171)	normalization-by-median.py -C 10 –n_hashes 4 –ksize 22 –hashsize 32e+8
Bignorm[b]	HeLa(SRR317049)	Bignorm -Q 10 -k 22 -m 512 -t 10
Diginorm[b]	HeLa(SRR317049)	normalization-by-median.py -C 10 –n_hashes 2 –ksize 22 –hashsize 32e+8

Weights in the Hela dataset

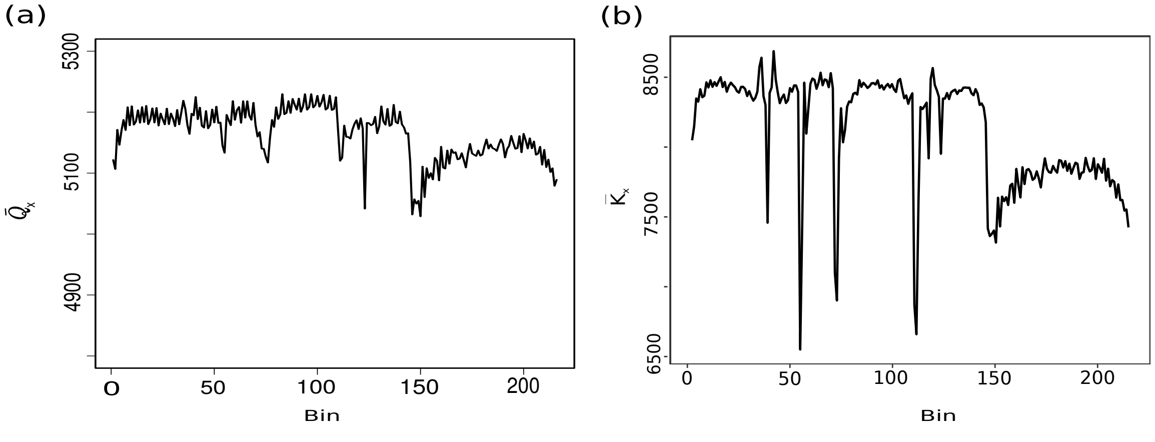

Figure S1: The position-wise distribution of (a) average phred quality based weight and (b) average label abundance weight in the HeLa dataset
